# Reducing Time to Discharge after Chemotherapy by Standardizing Workflow and Providing Outpatient Intravenous Hydration

**DOI:** 10.1097/pq9.0000000000000415

**Published:** 2021-06-23

**Authors:** Jitsuda Sitthi-Amorn, Allison Ast, Erin Harper, Brian Abbott, Yaser Alsaek, Wendy Bourland, Rachael Courtney, Arshia Madni, Aditya Sharma, Christopher Spencer, Lane McCurrach, Stacey Morgan, John McCormick, David Wittman, Liza-Marie Johnson

**Affiliations:** From the * Hospitalist Medicine Program, St. Jude Children’s Research Hospital, Memphis, Tenn.; † Department of Oncology, St. Jude Children’s Research Hospital, Memphis, Tenn.; ‡ PRA Health Sciences, Raleigh, N.C.; § Department of Nursing, St. Jude Children’s Research Hospital, Memphis, Tenn.; ¶ Division of Pediatric Nephrology, Department of Pediatrics, Le Bonheur Children’s Hospital, Memphis, Tenn.; ∥ Department of Pharmaceutical Services, St. Jude Children’s Research Hospital, Memphis, Tenn.; ** Office of Quality and Patient Care, St. Jude Children’s Research Hospital, Memphis, Tenn.

## Abstract

**Introduction:**

Patients receiving cyclophosphamide or ifosfamide chemotherapy require intravenous fluid hydration to prevent hemorrhagic cystitis. In selected patients without medical contraindications (ie, excess nausea/vomiting), this hydration may be completed after discharge. We aimed to reduce the time to discharge after completing mesna in patients receiving cyclophosphamide or ifosfamide therapy on an inpatient chemotherapy service.

**Methods:**

The quality improvement team performed a medical record review to capture the time to discharge after mesna therapy and the readmission rate and used quality improvement methods to redesign discharge workflow and increase patient involvement with the discharge process.

**Results:**

From August 2017 through July 2018, there were 160 admission encounters (73 patients) for cyclophosphamide or ifosfamide on a dedicated chemotherapy service. Of those encounters, 89 (55.6%) were appropriate for outpatient hydration; 48 (53.9%) of these encounters involved a patient who elected to receive outpatient hydration. Although the median time to discharge for the whole cohort did not change, in encounters where patients chose intravenous outpatient hydration, the median time to discharge was reduced from 2.82 to 0.66 hours (76.6% reduction) after implementing the new discharge workflow. No patients experienced readmission within 48 hours.

**Conclusions:**

Discharge workflow redesign and standardization reduced the time to discharge after chemotherapy in patients who chose outpatient hydration. Outpatient intravenous hydration after cyclophosphamide or ifosfamide appears safe and feasible in selected patient populations.

## Abstract 2

Supplemental Digital Content is available in the text.

## INTRODUCTION

**Fig. 1.**
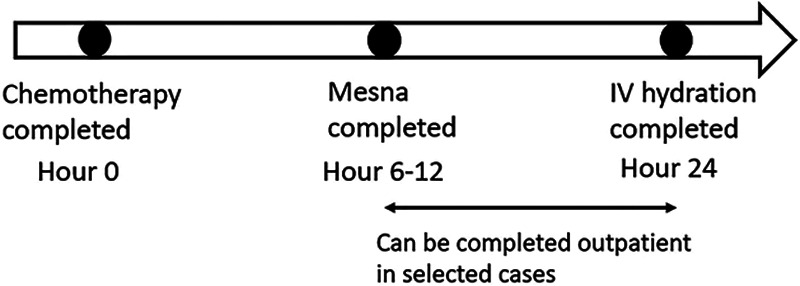
A diagram showing the timeline of mesna and intravenous hydration after cyclophosphamide or ifosfamide.

Patients receiving specific cyclophosphamide doses (>1,000 mg/m^2^) or any dose of ifosfamide require mesna and intravenous hydration to prevent hemorrhagic cystitis, a noninfectious cystitis characterized by gross hematuria that is a well-documented side effect of these drugs.^1,2^ This side effect is caused by the accumulation of toxic metabolites of chemotherapy in the bladder wall.^1,2^ Mesna prevents hemorrhagic cystitis neutralizing the toxic metabolites, and intravenous hydration promotes frequent urination, decreasing the contact time of the toxic metabolite with urinary bladder linings.^1,2^ Per local institutional protocol, the last dose of mesna is administered intravenously between 6 and 12 hours after the final dose of cyclophosphamide or ifosfamide, followed by posthydration fluids for a total of 24 hours after the last dose of chemotherapy (Fig. 1). Completing some part of the posttherapy hydration as an outpatient will reduce inpatient stays by up to 18 hours per admission. Early hospital discharges improve hospital flow and reduce wait time from the emergency department to inpatient beds.^3,4^ They also reduce cost and resource use.^5^ In children with cancer, the process to ensure timely, efficient, and safe discharge is intricate due to the complex nature of the diagnosis. Discharge planning requires involvement from multidisciplinary team members and patients and their families.^6^ Previous research has demonstrated that predischarge strategies, including goal identification,^7,8^ process redesign and standardization,^7–11^ improving communication among team members,^7,8,12,13^ and family involvement,^6,14^ lead to improved discharge efficiency.

To fully understand the elements of a chemotherapy discharge and the barriers to timely discharge, a multidisciplinary team was formed to identify steps to reduce discharge time, thereby increasing bed availability and improving the flow of scheduled and unscheduled admissions. The quality improvement team chose to focus on patients receiving cyclophosphamide or ifosfamide because early discharge with outpatient hydration in this group would have the most significant impact on the length of stay among patients routinely admitted for chemotherapy. The aim was to reduce the median time to discharge from 8.7 to 2 hours after completion of mesna in patients receiving cyclophosphamide or ifosfamide on chemotherapy service within 12 months.

## METHODS

### Context

St. Jude Children’s Research Hospital is a free-standing 73-bed pediatric hospital specializing in treating children with cancer and other catastrophic illnesses. It has approximately 4,300 inpatient admissions per year. For nonlocal patients, the hospital provides housing within 3 miles of the main campus. An outpatient infusion and acute care center are available to provide supportive care around chemotherapy delivery, including outpatient intravenous fluid administration. Patients admitted for routine chemotherapy are admitted to the chemotherapy service, staffed by a team of rotating pediatric oncology hospitalist physicians and clinical pharmacists. The hospitalists manage routine chemotherapy admissions and work closely with the patients’ primary oncologists on any complicated issues and discharge planning.

Patients admitted for cyclophosphamide or ifosfamide chemotherapy can choose to complete intravenous hydration as an inpatient or outpatient. Patients who choose to complete outpatient hydration are connected to a bag of fluid delivered via a continuous ambulatory delivery device before discharge and return to the outpatient clinic the next day for disconnection. Parents or family members must complete training on the continuous ambulatory delivery device pump alarm systems by nurse educators. The caregivers have to correctly teach-back before being signed off as competent. Each patient must have a caregiver of documented competency in the medical record before getting outpatient intravenous hydration.

At the start of the project in August 2017, the hospital administration identified an improvement in bed turnaround times crucial for hospital flow to prevent routine chemotherapy admission cancelations due to lack of bed availability. The chemotherapy service workflow, including the rounding and discharge processes, was not standardized. Discharge tasks (**Table 1, Supplemental Digital Content 1**, which displays discharge criteria and predischarge tasks for the safe discharge of patients receiving cyclophosphamide or ifosfamide chemotherapy, http://links.lww.com/PQ9/A264), including discharge medication reconciliation, home intravenous fluid teaching, and routine discharge teaching, had no definitive timeline for completion. Few physicians offered outpatient intravenous hydration, and many were unaware that outpatient hydration was feasible for patients receiving cyclophosphamide or ifosfamide chemotherapy.

### Interventions

**Fig. 2.**
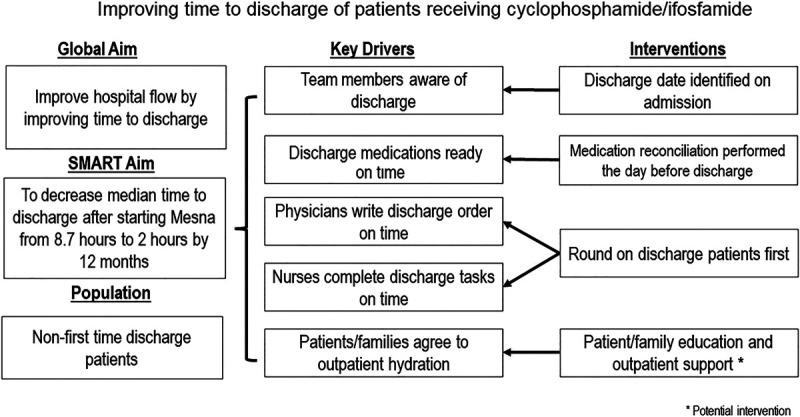
Key driver diagram demonstrating key drivers, interventions, and potential interventions for the efficient discharge.

A quality improvement team composed of key stakeholders involved in the discharge process was formed. Team members included oncology hospitalist physicians, an advanced practice provider, a clinical pharmacist, nurses, an educator, a bed manager, and a quality improvement project coach. The team identified the discharge process, tasks required before discharge (**Table 1, Supplemental Digital Content 1**, which displays discharge criteria and predischarge tasks for the safe discharge of patients receiving cyclophosphamide or ifosfamide chemotherapy, http://links.lww.com/PQ9/A264), key drivers necessary for timely discharge, and potential interventions (Fig. 2).

**Fig. 3.**
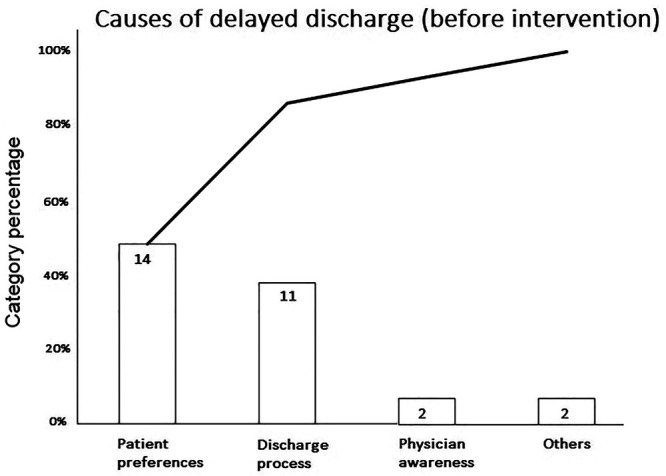
Pareto diagram showing causes of delayed discharge before the intervention.

The quality improvement team conducted a process observation by reviewing the electronic medical record and interviewing physicians and nurses who treated the patients who had delayed discharge after completion of cyclophosphamide or ifosfamide chemotherapy. Causes of delay included the patient’s preference for inpatient intravenous hydration (48.3%), delayed discharge processing (37.9%), physicians being unaware of outpatient hydration (6.9%), and other causes (6.9%) (Fig. 3). The process observation confirmed the result of informal interviews with patients and family members, which showed that about half were uncomfortable being outpatient while receiving intravenous fluids.

At the time of the project, the project team lacked the resources to address patient preference for inpatient hydration; therefore, the team decided to optimize the discharge process. Delays in the discharge process were multifactorial. For example, preparing a patient’s discharge medications could require 3 hours. Pharmacists could not start preparing medication until both physicians and clinical pharmacists performed discharge medication reconciliation. Bedside nurses often started discharge teaching only after receiving verbal confirmation of the same-day discharge from physicians, which occurred after daily rounding.

**Fig. 4.**
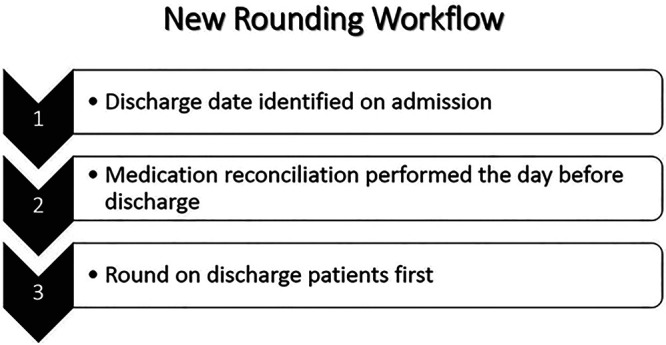
New discharge rounding workflow.

Based on these findings and the fact that several stakeholders can complete many of the necessary tasks only after medication reconciliation is completed and before the final discharge decision, the quality improvement team designed a new discharge workflow for the chemotherapy service: (1) identify tentative discharge date on the day of admission by inpatient attending physician at the time of admission order review; (2) complete discharge medication reconciliation the day before discharge, and prepare discharge medications the day before discharge; and (3) round on discharge patients first on the morning of discharge (Fig. 4). Upon admission to the chemotherapy service, physicians would write the identified discharge dates on the census board so that the information would be accessible to nurses and bed managers. Change concepts for the interventions are standardization (standardizing the rounding workflow), give people access to information (communicating discharge date to the clinical care team), find and remove bottlenecks (by preparing discharge medication before the day of discharge), and do tasks in parallel (by rounding on discharge patients first so that nurses can start performing nursing discharge tasks).^15^

### Study of the Interventions

During each plan-do-study-act cycle to test the intervention, the team asked oncology hospitalist physicians to complete a short written questionnaire to determine whether the new process negatively affected the workflow. Their responses were analyzed to evaluate the effect of the intervention and incorporated into process adaptation.

### Measures

The quality improvement team tracked the time to discharge after mesna therapy of patients receiving cyclophosphamide or ifosfamide chemotherapy throughout the project. Mesna administration time was retrieved from when the inpatient nurse scanned mesna into the electronic record just before administration. There was no record of the actual time of completion; however, this is a short infusion of approximately 15 minutes. Bedside nurses entered the discharge time when they completed all discharge tasks, and the patients left the hospital. The balancing measure was the readmission rate within 48 hours of discharge. The quality improvement team decided to use this shorter readmission window instead of the commonly used 7-day readmission rate^16^ because many patients are admitted for fever and neutropenia within seven days from completion of cyclophosphamide or ifosfamide.^17^

### Analysis

Time to discharge from each encounter was plotted on run charts. The centerline was shifted based on established rules for special cause variation in the context of additional interventions.^18,19^

The hospital’s Institutional Review Board approved this project as a quality improvement project and waived the written informed consent.

## RESULTS

From August 2017 through July 2018, there were 160 chemotherapy service admission encounters (73 patients) for cyclophosphamide or ifosfamide. Patients were candidates for outpatient hydration in 89 encounters (50 patients). Noneligible patients for early discharge were excluded (n = 70). The exclusion criteria for early discharge included: care completed overnight (n = 44), unresolved medical concerns such as uncontrolled emesis (n = 17), or first-time chemotherapy discharge requiring dedicated teaching (n = 10). Patients chose outpatient hydration in 48 (53.9%) of the 89 encounters included in the analysis.

**Fig. 5.**
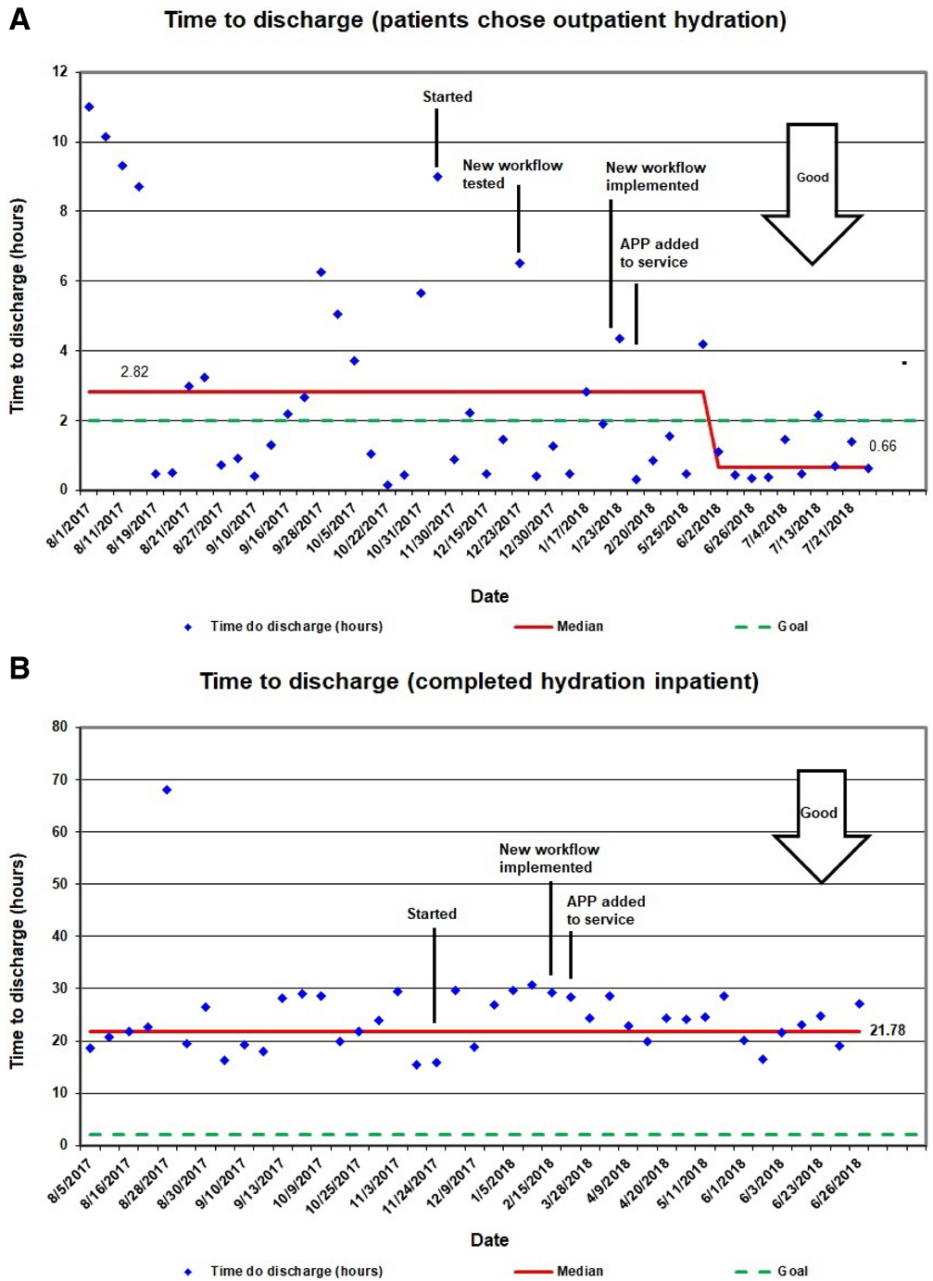
Time to discharge. Run chart showing time to discharge after completion of cyclophosphamide or ifosfamide for patients that chose outpatient intravenous hydration (A) and patients that completed intravenous hydration inpatient (B).

The median time to discharge after completion of mesna for patients who chose outpatient hydration improved from a baseline of 2.82 to 0.66 hours (Fig. 5A). However, the median time to discharge did not change for the whole cohort (9.73 hours) and for patients who chose inpatient hydration (21.78 hours) (Fig. 5B). Three patients in our study initially chose to complete inpatient posthydration therapy and later chose outpatient hydration. On the other hand, no patient initially receiving outpatient hydration switched to inpatient hydration. No physician reported any negative impact of the new rounding workflow.

An essential contextual element unrelated to this project, but likely benefitting time to discharge, was the addition of a full-time advanced practice provider to the chemotherapy service in March 2018, which helped by providing continuity and consistent workflow.

## DISCUSSION

The discharge process for patients admitted for chemotherapy is complex and requires collaboration between multidisciplinary team members.^6^ Using quality improvement methodology, the quality improvement team successfully reduced the time to discharge for patients admitted for cyclophosphamide or ifosfamide chemotherapy who were comfortable with outpatient intravenous hydration, demonstrating how a simple workflow redesign can benefit hospital flow.

Like previously published studies, the quality improvement team reduced hospital discharge time using process redesign and standardization.^7–11^ However, when the team first launched the project, the aim was to reduce the overall baseline discharge time to two hours after mesna. To achieve that, most patients would have to leave the hospital on outpatient intravenous hydration. Still, the team learned that some patients and family members were not comfortable with outpatient hydration. Due to time and resource limitations, the team decided not to explore causes and interventions for patients who were uncomfortable with outpatient hydration. Several tools and interventions to help understand and improve patients’ comfort level with self-management include shared decision-making models,^20,21^ motivational interviewing,^22,23^ face-to-face support,^24^ and supervised practice.^25^ Some of these interventions could be tested and explored in future quality improvement projects. However, patients who choose outpatient hydration do so because they do not like to stay in the hospital, and the team successfully discharged them sooner.

This project had certain limitations. First, for patients who chose outpatient hydration, the time points for the completion of care were when they received the last dose of mesna. However, for patients who chose inpatient hydration, the time points when they were considered ready for discharge were variable. These variations were not measured and may explain why no reduction in time to discharge occurred in the latter group. To better track time to discharge, the quality improvement team worked with the hospital information service team to develop a mandatory “time care completed” tab for bedside nurses to enter once they complete all necessary medical tasks, and patients are ready for discharge. This change will allow accurate monitoring of time to discharge in future projects. Moreover, the quality improvement team did not address patient preference for outpatient hydration, which is the most important key driver for timely discharge. Finally, the hospital provided housing close to the main campus for nonlocal patients, making complex outpatient care feasible. However, outpatient intravenous hydration may not be possible for centers without local accommodation.

Nevertheless, by using a simple workflow redesign, we shortened the time to discharge for patients who chose outpatient hydration without increasing readmission and have incorporated the new workflow in new employee orientation to ensure sustainability. Further research incorporating patient-centered healthcare tools such as motivational interviewing is needed to address outpatient intravenous hydration barriers from patients’ and families’ perspectives.

## DISCLOSURE

Dr Abbot participated in the quality improvement project while he worked at our institution and is currently a PRA health sciences employee. The other authors have no financial interest to declare in relation to the content of this article.

## ACKNOWLEDGMENTS

The authors thank Patricia Flynn, MD, Ibrahim Qaddoumi, MD, and Windy Fitzhugh, PNP, for designing the interventions and Cherise Guess, Ph.D., ELS, for her assistance in editing the article.

## Supplementary Material


