# Supplementary material for: Reducing Time to Discharge after Chemotherapy by Standardizing Workflow and Providing Outpatient Intravenous Hydration
Source: Pediatr Qual Saf. 2021 Jun 23;6(4):e415. doi: 10.1097/pq9.0000000000000415 (PMC8225375; doi:10.1097/pq9.0000000000000415)
Supplement: Supplementary file 1 [file pqs-6-e415-s001.pdf]

Supplemental Table 1: Discharge criteria and pre-discharge tasks for the safe discharge of patients receiving cyclophosphamide or ifosfamide chemotherapy

Criteria and tasks	Required for all patients	Required for patients discharged with outpatient intravenous hydration
Clinical criteria		
-Clinically stable	Yes	Yes
-No uncontrolled nausea/vomiting	Yes	Yes
-Patient/family comfortable with IV outpatient hydration	No	Yes
Discharge medication and fluids		
-Medication reconciliation planned by physicians	Yes	Yes
-Medication reconciliation reviewed by pharmacists	Yes	Yes
-Discharge medication ready	Yes	Yes
-Discharge intravenous fluid ready	No	Yes
Education and teaching		
-Discharge teaching by bedside nurse	Yes	Yes
-Outpatient IV fluid teaching	No	Yes
Miscellaneous		
-Discharge orders entered	Yes	Yes
-Transportation ready	Yes	Yes
